# DIGITAL AGEISM PERSISTS IN ARTIFICIAL INTELLIGENCE-GENERATED IMAGES

**DOI:** 10.1093/geroni/igae098.4162

**Published:** 2024-12-31

**Authors:** Nicole Derenne, Lindsey Martens, Nicole Virgin, Phillip Hoffarth, Richard Van Eck, Marilyn Klug, Gunjan Manocha, Donald Jurivich

**Affiliations:** University of North Dakota, Grand Forks, North Dakota, United States; University of North Dakota, Grand Forks, North Dakota, United States; University of North Dakota, Grand Forks, North Dakota, United States; University of North Dakota, Grand Forks, North Dakota, United States; University of North Dakota, Grand Forks, North Dakota, United States; University of North Dakota, Grand Forks, North Dakota, United States; University of North Dakota, Grand Forks, North Dakota, United States; University of North Dakota, Grand Forks, North Dakota, United States

## Abstract

Artificial intelligence systems (AI), including text-to-image models, often reflect and amplify societal age-related biases, a phenomenon known as digital ageism. This study examines if instances of digital ageism in AI-generated representations of older adults lessen over time. Examining how digital ageism evolves in AI-image generation can provide insight into the interplay between technology advancements, societal attitudes toward aging, and the well-being of older adults interacting with the dynamic digital landscape. This mixed methods project compared 164 images generated by Open AI’s DALL-E 2 image generator at two points, one year apart. Text prompts, identical at both points of inquiry, included terms from the geriatric lexicon (e.g., frail older adult, dementia, successful aging). Images were qualitatively evaluated to identify perceived gender, race, socioeconomic status, and emotional expression. Quantitative analysis of the frequency with which each category was represented found significant differences in gender and socioeconomic representation at both points of inquiry but no difference between the two points. The number of white racialized representations of older adults was 5-fold higher than other racialized representations at both points, with an increase of Asian racialized representations at the second point of inquiry. There was no significant difference between emotional expressions between the inquiry points. Despite the increased social emphasis on positive views on aging, AI text-to-image generators persistently generated predominantly white racialized middle-class male figures. A limitation of this study is that it focuses only on AI text-to-image generation and no other AI-generated content.

